# Serotype and clonal distribution dynamics of invasive pneumococcal strains after PCV13 introduction (2011-2016): Surveillance data from 23 sites in Catalonia, Spain

**DOI:** 10.1371/journal.pone.0228612

**Published:** 2020-02-06

**Authors:** Guillermo Ludwig, Selene Garcia-Garcia, Miguel Lanaspa, Pilar Ciruela, Cristina Esteva, Mariona Fernandez de Sevilla, Alvaro Diaz-Conradi, Carmina Marti, Montse Motje, Carme Galles, Montse Morta, Conchita Izquierdo, Fernando Moraga-Llop, Magda Campins, Luis Salleras, Mireia Jane, Angela Dominguez, Juan Jose Garcia-Garcia, Carmen Muñoz-Almagro, and Catalan Study Group of Invasive Pneumococcal Disease

**Affiliations:** 1 Institut de Recerca Sant Joan de Deu, Hospital Sant Joan de Deu, Barcelona, Spain; 2 CIBER de Epidemiologia y Salud Publica (CIBERESP), Instituto de Salud Carlos III, Madrid, Spain; 3 Agencia de Salut Publica de Catalunya (ASPCAT), Generalitat de Catalunya, Barcelona, Spain; 4 Hospital de Nens, Barcelona, Spain; 5 Hospital General de Granollers, Granollers, Spain; 6 Hospital Universitari de Girona Dr. Josep Trueta, Girona, Spain; 7 Hospital Sant Jaume, Calella, Spain; 8 Hospital Sant Joan de Deu de Manresa, Barcelona, Spain; 9 Hospital Universitari Vall d'Hebron, Barcelona, Spain; 10 Grup de Recerca en Epidemiologia i Salut Pública, Vall d'Hebron Institut de Recerca, Barcelona, Spain; 11 Departament de Medicina, Universitat de Barcelona, Barcelona, Spain; 12 Departament de Medicina, Universitat Internacional de Catalunya, Barcelona, Spain; Universidad Nacional de la Plata, ARGENTINA

## Abstract

**Background:**

The objective of this study is to describe incidence and shifts of serotype and clonal distribution of invasive *Streptococcus pneumoniae* strains in four different age groups (<5 years, 5–17 years, 18–64 years and >65 years) during a period of intermediate PCV13 vaccination coverage (2011–2016) in Catalonia, Spain.

**Methods:**

We included all pneumococcal strains systematically sent to the Catalan support laboratory for molecular surveillance of invasive pneumococcal disease (IPD) located at Hospital Sant Joan de Deu, Barcelona. Two study periods were considered: 2011–13, early PCV13 vaccination period (EVP) and 2014–2016, late vaccination period (LVP).

**Results:**

A total of 2142 strains were included in the study. Five years after intermediate introduction of PCV13 in our population, a significant decrease of overall incidence of IPD in children <5 years was observed (incidence rate ratio 0.5, 95% confidence interval 0.4–0.8). However, in seniors older than 65 years, a significant increase of overall incidence of IPD was observed (IRR 1.4, 95% CI 1.1–1.7). The contribution of PCV13 vaccine serotypes to IPD declined significantly in all age groups: from 59% to 38.1% in <5 years; 82.7% to 59% in 5–17 years, 47.8% to 34.1% in 18–64 years and 48.2% to 37% in >65 years. Results found when comparing both periods were consistent with IRRs observed year by year. In children <5 years, the three major serotypes detected were 1, 24F and 19A in EVP vs 24F, 14 and 10A in LVP. Among patients 5–17 years the first three serotypes were 1, 12F and 14 both in EVP and LVP. Among adults 18–64, the three major serotypes detected were 1, 12F and 8 vs 8, 12F and 3, respectively. Finally, in patients >65 years the most frequently isolated serotypes were 3, 19A and 7F vs 3, 14 and 12F, respectively. Regarding clonal complexes (CCs) expressing mainly PCV13 serotypes, significant decreases of the proportions of CC306, CC191 and CC320 were observed, while CC156 showed a significant increase. As for CCs expressing mostly non-PCV13 serotypes, significant increases in ST989, CC53 and CC404 were showed.

**Conclusions:**

Despite low vaccine coverage in our setting a significant decrease of incidence of IPD was observed in children younger than 5 years. The modest indirect protection against vaccine serotypes causing IPD in elderly indicate the need for the inclusion of more serotypes in future high-valent PCV and vaccinating old adults should be considered.

## Introduction

*Streptococcus pneumoniae* is a commensal microorganism of the human nasopharynx [1] but is also responsible for significant morbidity and mortality worldwide especially affecting children under 5 years and adults over 65. Many of these deaths could be prevented by vaccination. The most severe form is the invasive pneumococcal disease (IPD), which includes pneumonia, meningitis and septicemia [2]. There are more than 95 different serotypes of *S*. *pneumoniae* [3], several of them causing invasive disease. In 2000, a protein-polysaccharide conjugate vaccine against seven serotypes (PCV7) was licensed in the USA. PCV7 was introduced in Spain in 2001. Due to an increasing relevance of non-vaccine serotypes new vaccines were developed [4]; PCV10 (PCV7 plus 1, 5 and 7F), and PCV13 (PCV10 plus 3, 6A and 19A). PCV10 and PCV13 were introduced in Spain in 2010. However, PCV13 was mainly used in children younger than 5 years with less than 5% of PCV10 used [5]. Because pneumococcal vaccines were not subsidized by the Public Health Service in Catalonia until 2016 (except for children with risk factors), PCV13 coverage among under 5 year children in Catalonia was estimated at 55% in 2012–2013 [5], 63.6% in 2012–2016 [6] and 78% in 2015 [7]. Vaccination against pneumococcal disease with PCV13 follows the 3+1 schedule in Catalonia, corresponding to 3 doses in the first 6 months of life (at 2, 4 and 6 months old) followed by a booster dose at 12 to 15 months old.

PCV13 proved effective in preventing pediatric pneumococcal disease and in decreasing nasopharyngeal carriage of the vaccine serotypes [6, 8–13] as did PCV7 before it [14–17]. Vaccinating children with PCV13 also prevents IPD in adult patients and non-vaccinated children through indirect effects (herd immunity) by interrupting transmission of *S*. *pneumoniae* [6, 8–13]. Despite this success, concerns about impact on overall IPD incidence remain. Concretely, despite PCV13 introduction, vaccine failure against serotype 3 [6, 11, 13, 18, 19] and a rise of serotype 19A [20–21] have been observed. Moreover, the emergence of pediatric and adult IPD caused by non-vaccine serotypes is still a major challenge in order to overcome the burden of disease [22–23], and warrants a continuous molecular surveillance enabling an early assessment of candidate serotypes to be prioritized in future PCVs.

Pneumococcal surveillance should not be limited to characterizing serotypes because we can miss important population shifts at the genetic level. Describing the dynamics of clonal types is essential to fully understand the potential and ability of pneumococcus to elude vaccine pressure.

The objective of this study was to characterize the dynamics of serotypes distribution and clonal composition of *S*. *pneumoniae* isolates causing IPD in four different age groups (<5 years, 5–17 years, 18–64 years and >65 years) during a period of intermediate PCV13 vaccination coverage (2011–2016) in Catalonia, Spain.

## Materials and methods

### Study setting and design

We conducted a prospective study which included pneumococcal invasive isolates received at the University Hospital Sant Joan de Deu Barcelona between 2011 and 2016. In 2009, the Molecular Microbiology Laboratory was designated by the government of Catalonia, Spain, as reference laboratory for molecular surveillance of IPD. Catalan Hospitals are invited, not forced, to send invasive pneumococcal strains. Among them, there were 23 that systematically sent isolates to the reference laboratory. They are committed to this task as members of Catalan Study Group of Invasive Pneumococcal Disease. In this study, we only considered those 23 that systematically sent samples during the study period.

In Catalonia, with a population of 7.5 million estimated for 2016, the catchment population of the health facilities included in the study captured 44% of the Catalan population and, specifically, 67% of children younger than 5 years. These proportions were estimated using public records for each hospital, where age dependent patient’s admissions data was registered (“Conjunt minim basic de dades”, www.catsalut.gencat.cat). We then applied these proportions to the total population and the population by age according to municipal registries that are updated yearly (“Padró municipal d’habitants”, www.idescat.cat).

Comparing the first year of the study (2011) vs the last one (2016), the reference population of the 23 health facilities varied between 256.965 and 231.925 children younger than 5 years, 551.803 and 526.256 old children between 5–17 years, 1.669.954 and 1.632.967 adults from 18–64 years and 422.498 and 458.573 seniors older than 65 years.

### Ethics statement

This study was approved by the ethics committee of Hospital Sant Joan de Déu (CEIm Fundació Sant Joan de Déu; Internal code: PIC-101-19. Written consent) on data protection and law 14/2007, on July 3rd, on Biomedical Research.

For the present study, no informed consent was requested as this is a molecular epidemiology surveillance-based study in which samples were duly anonymized.

### Definitions

IPD was defined as the presence of clinical findings of infection confirmed by isolation of *S*. *pneumoniae* by culture in any sterile fluid. IPD was classified according to International Classification of Disease (ICD-10), specific for diseases caused by *S*. *pneumoniae* including occult bacteraemia/sepsis, meningitis, pneumonia, parapneumonic empyema, peritonitis and arthritis.

We stratified the study population in four age groups: younger children less than 5 years of age; older children between 5–17 years; adults aged 18–64 years; and seniors older than 65 years.

### Microbiological identification and serotyping

Isolates were identified in the different health facilities by standard microbiological techniques that included Gram staining, optochin sensitivity test and bile solubility test. After this presumptive identification, strains were delivered to the Molecular Microbiology Department of the University Hospital Sant Joan de Deu. On arrival at the laboratory, identification of capsular pneumococcal serotypes was performed using multiplex PCR combined with fragment analysis and automated fluorescent capillary electrophoresis. This technique has been used since 2010 and allows the detection of 40 serotypes/serogroups: (1, 2, 3, 4, 5, 6A/6B, 6C, 7F/7A, 7C/(7B/40), 8, 9V/9A, 9N/9L, 10A, 10F/(10C/33C), 11A/11D/11F, 12F/(12A/44/46), 13, 14, 15A/15F, 15B/15C, 16F, 17F, 18/(18A/18B/18C/18F), 19A, 19F, 20, 21, 22F/22A, 23A, 23B, 23F, 24/(24A/24B/24F), 31, 33F/(33A/37), 34, 35A/(35C/42), 35B, 35F/47F, 38/25F and 39) [24]. All strains were also sent to the National Pneumococcus Reference Center of Majadahonda, Madrid, Spain, to complete serotyping by Quellung reaction.

### Clonal analysis

Clonal analysis was performed with multilocus sequence typing (MLST). MLST was performed as reported elsewhere [25]. The assignment of alleles and sequence types (ST) was carried out using the software at the web site http://pubmlst.org/spneumoniae/. Analysis of ST and assignment to clonal complexes (CCs) were performed with the eBURST program. STs that shared six out of seven allelic variants (single locus variants) and five out of seven allelic variants (double locus variants) were considered a CC.

### Statistical analysis

We used Chi-square or Fisher’s exact tests to compare proportions. Incidence rates of IPD were calculated assuming constant population during a given year. We divided the study period in two categories: early vaccine period (EVP) spanning from 2011 to 2013, and late vaccine period (LVP) from 2014 to 2016 for analysing changes in specific serotypes and clones. Cumulative incidences were compared using the Chi-square test with the rate ratio. Incidence rate ratios (IRRs) were estimated by dividing observed over expected rates in 2011 and calculated for each age-serotype group. Year 1 to 5 represents years encompassed from 2012 to 2016. We calculated 95% confidence intervals (CI) and 2-sided p-values < 0.05 were considered to be statistically significant. Statistical analyses were performed using SPSS for Windows, version 17.0 (SPSS).

## Results

A total of 2142 episodes of IPD, occurred in 2085 patients, with serotype and clonal data were included during the study period (2011–2016). Over half of the episodes occurred in males (1246; 58.2%). The median age of the patients was 60.9 (IQR = 41.8) years (range 1 day—104 years old). Three hundred fifty seven (16.7%) of the strains were isolated in younger than 5 years, 91 (4.25%) in 5–17 years, 768 (35.85%) in 18–64 years, and 926 (43.2%) in older than 65 years. Most cultures were performed in blood (1912; 89%), followed by cerebrospinal fluid (108; 5%), and pleural fluid (93; 4%).

### Proportion and incidence rates of IPD episodes in early and late vaccine period

**Fig 1:**
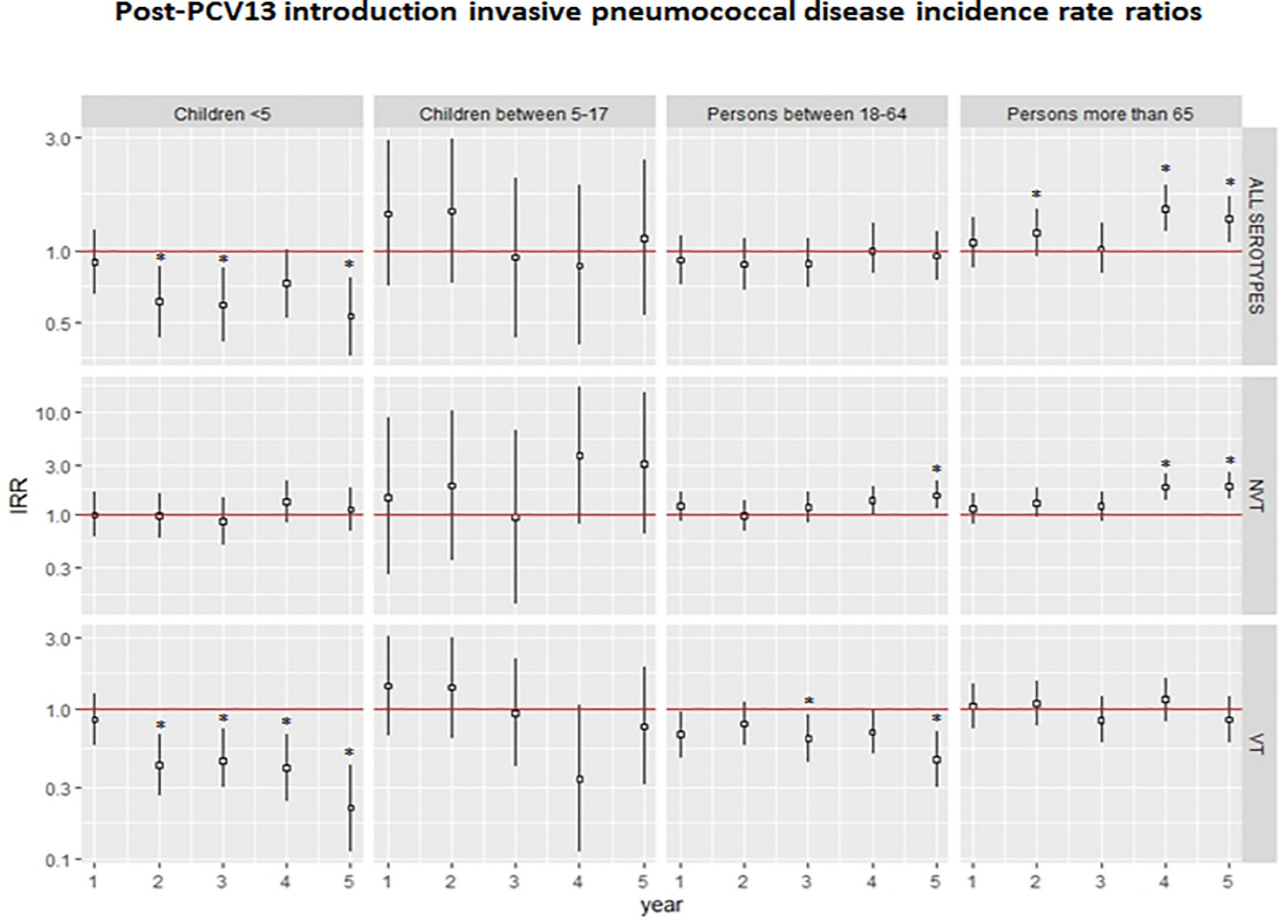
Post-PCV13 introduction invasive pneumococcal disease IRRs. Data are pooled IRRs. IRRs were estimated by comparing observed incidence (in each of the years 2012 to 2016) to expected (incidence in 2011) for each age-serotype group. 95% CIs presented on a logarithmic scale by error bars. Year 1 to 5 represents years encompassed from 2012 to 2016.

Five years after intermediate introduction of PCV13 in our population (Fig 1), a significant decrease of overall incidence of IPD in children <5 years was observed (IRR 0.5, 95% CI 0.4–0.8). However, in seniors older than 65 years, a significant increase of overall incidence of IPD was observed (IRR 1.4, 95% CI 1.1–1.7). Overall IRRs were calculated comparing incidence rate in 2016 to 2011. Comparing EVP and LVP, the highest IPD incidence was among elderly, and the lowest among 5–17 age group (Table 1).

**Table 1:** Proportion and incidence rates of IPD episodes in EVP and LVP.

Age groups	EVP (%)	LVP (%)	*p-value*	EVP (IR*)	LVP (IR)	IRR (CI 95%)	*p-value*
<5	210 (19.74)	147 (13.64)	-	27.71	20.64	0.74 (0.60–0.92)	0.006
5–17 18–64	52 (4.89) 387 (36.37)	39 (3.62) 381 (35.34)	- -	3.07 7.62	2.30 7.74	0.75 (0.49–1.14) 1.02 (0.89–1.17)	0.179 0.511
>65	415 (39.00)	511 (47.40)	-	32.48	38.46	1.18 (1.04–1.35)	0.010
**IPD caused by PCV13 serotypes**							
<5	124 (59.05)	56 (38.10)	<0.001	16.36	7.86	0.48 (0.35–0.66)	<0.001
5–17 18–64	43 (82.69) 185 (47.80)	23 (58.97) 130 (34.12)	<0.001 <0.001	2.54 3.64	1.35 2.64	0.53 (0.32–0.88) 0.73 (0.58–0.91)	0.014 0.005
>65	200 (48.19)	189 (36.99)	<0.001	15.65	14.22	0.91 (0.74–1.11)	0.345
**IPD caused by non-PCV13 serotypes**							
<5	86 (40.95)	91 (61.91)	<0.001	11.35	12.78	1.13 (0.83–1.50)	0.476
5–17 18–64	9 (17.31) 202 (52.20)	16 (41.03) 251 (65.88)	0.015 <0.001	0.53 3.98	0.94 5.10	1.77 (0.79–4.2) 1.28 (1.07–1.55)	0.170 0.008
>65	215 (51.81)	322 (63.01)	<0.001	16.83	24.23	1.44 (1.21–1.71)	<0.001

IPD, Invasive pneumococcal disease; EVP, Early vaccine period; LVP, Late vaccine period; IR, Incidence rate per 100.000 person-year; IRR, Incidence rate ratio, CI, Confidence interval.

*Incidence: episodes per 100.000 person-year living in the reference area of 23 hospitals according to data from “Catalonian Institute of Statistics” (www.idescat.net).

The incidence of IPD caused by serotypes included in PCV13 decreased significantly among young children (RR 0.48; 95% CI 0.35–0.66), older children (RR 0.53; 95% CI 0.32–0.88) and adults aged 18–64 (RR 0.73; 95% CI 0.58–0.91). No significant variations in IPD incidence rates caused by vaccine serotypes were showed in seniors older than 65 years. Conversely, a significant incidence increase of non-PCV13 serotypes was only detected in adults (RR 1.28; 95% CI 1.07–1.55) and seniors (RR 1.44; 95% CI 1.21–1.71). Both in young and older children, non-significant variations between EVP and LVP were observed. Results found when comparing both periods of three years each were consistent with IRRs observed year by year, as shown Fig 1.

### Serotype distribution and incidence rates of invasive serotypes in EVP and LVP

**Fig 2:**
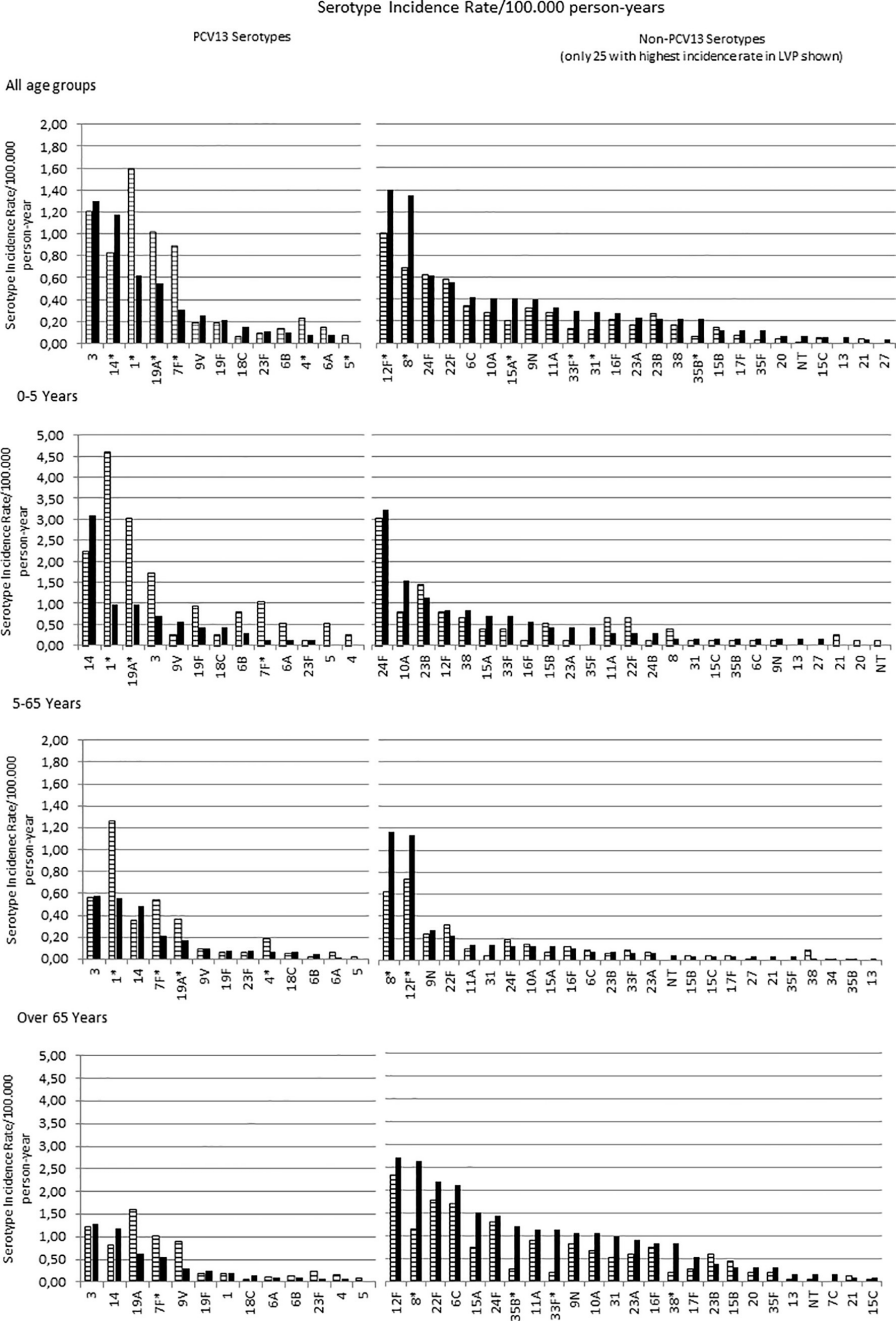
Serotype distribution and incidence rate/100.000 person-years of invasive serotypes during the study period. EVP, early vaccine period; LVP, late vaccine period; NT, non-typable. Filled bars depict EVP, while stripped ones LVP.* p<0.05 (Chi-square test). Despite in the text below we explain serotype distribution results by four age groups, because older children between 5–17 years are the smallest age group (it only includes 91 out of total 2142 IPD episodes), in Fig 2 patients aged from 5 to 65 years have been grouped. Hence, in this figure there are depicted three age groups instead of four.

We identified a total of 53 different serotypes causing IPD. The proportion of IPD episodes attributable to serotypes included in PCV13 dropped significantly in young children where PCV13 serotypes accounted for 59% of the IPD episodes in the EVP, and only 38% in the LVP (p<0.001). Three out of the five vaccine serotype pneumococcus that were more frequently isolated during EVP showed a significant decreased incidence in LVP (serotypes 1, 19A, 7F; Fig 2). Incidence of serotype 3 did not change, and serotype 14 was significantly more frequent in LVP. The incidence of non-vaccine serotypes increased during the study period, with 12F and 8 being the most emergent.

Serotype distribution varied substantially between age-groups, particularly concerning the non-PCV13 serotypes. In young children the most frequent serotypes in LVP were 24F, 10A and 23B, none of them with significant incidence variations. In older children, serotype 12F was the most frequent in EVP and also in LVP, with non-significant incidence variation. In adults aged 18–64, serotypes 8 and 12F were the most frequent both in EVP and LVP, showing a significant increase in incidence and clearly standing out as the dominant non-vaccine serotypes. Similarly, in seniors older than 65 serotypes 8 and 12F were the most frequent in LVP, but only serotype 8 showed a significant increased incidence. Incidence was more evenly distributed between different serotypes, with the top ten showing increased incidences, particularly serotypes 35B, 33F, and 38.

Overall, the incidence of five of the PCV13 serotypes significantly decreased during the study period, particularly serotype 1, 7F, and 19A, with serotype 5 disappearing during LVP.

**Fig 3:**
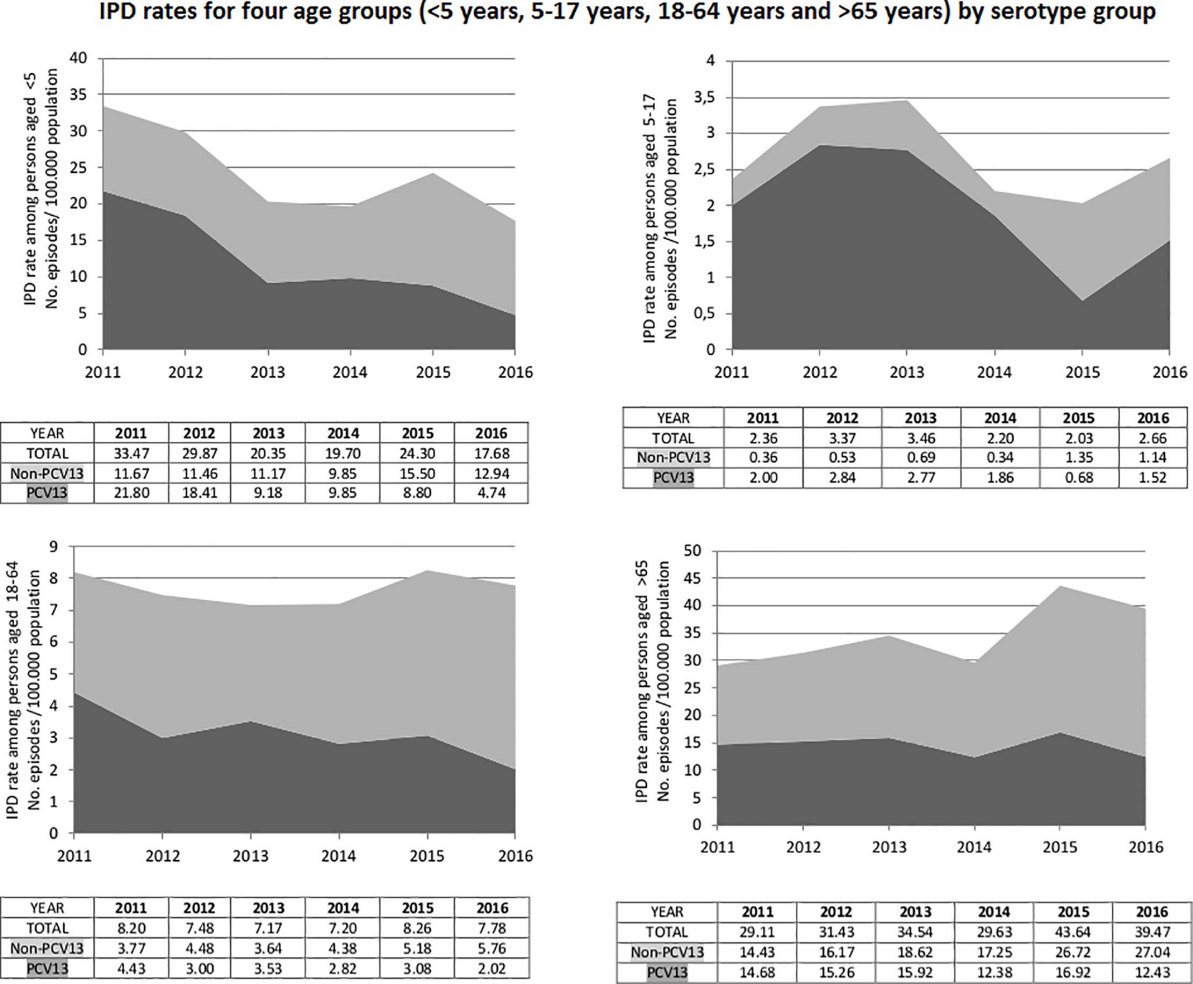
IPD rates for each of four age groups by serotype group.

These general trends in PCV13 serotypes’ incidence were quite similar by age-group with certain differences in predominant serotypes. The incidence of serotype 3 substantially decreased among young children (from 1.72 to 0.70 cases per 100.000 person-years, p = 0.084), making serotype 14 the outstanding leader in IPD caused by PCV13 serotypes. Among 5–17 years group, despite its significant incidence decrease (from 2.07 to 0.65 cases per 100.000 person-years, p<0.001), serotype 1 remained a frequently isolated PCV13 serotype in IPD cases together with serotype 14. In adults group aged 18–64, serotype 1 remained as the main PCV13 serotype causing IPD in EVP (from 0.87 to 0.49 cases per 100.000 person-years, p = 0.02) followed by serotype 3 with non-significant incidence changes. The particularity in older than 65 years is that the most frequent serotypes causing IPD in LVP remain PCV13 serotypes, namely serotypes 3 and 14, while in the other age-groups there was a consistent increase of non-vaccine PCV13 serotypes as the top serotypes causing IPD. Fig 3 shows IPD rates for each of four age groups by serotype group.

### Clonal distribution of isolates

**Fig 4:**
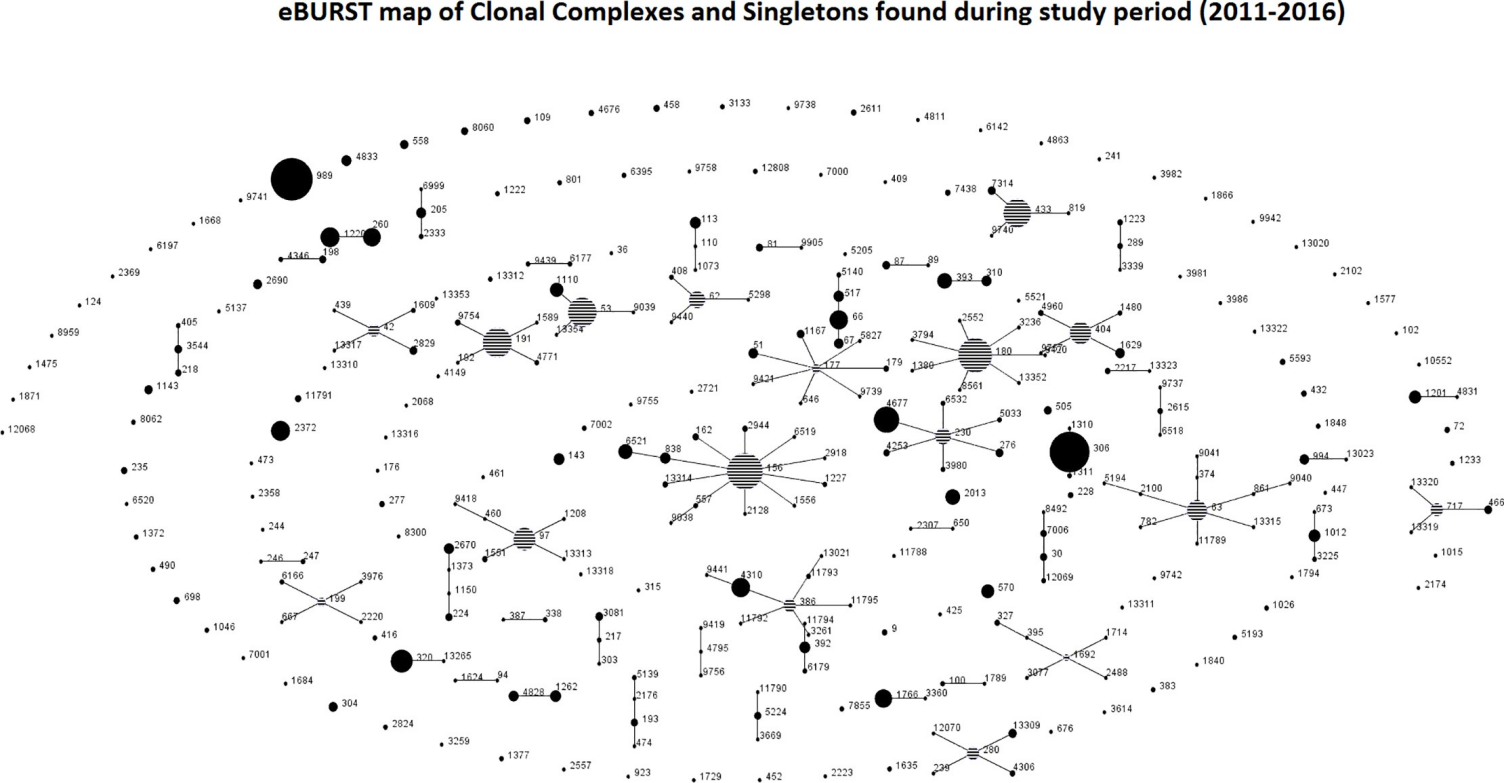
eBURST map of clonal complexes (CCs) and singletons found during study period (2011–2016).

Among the 2142 IPD isolates a total of 307 different sequence type (ST) were identified (Fig 4). Around 60% of the ST were grouped in CCs (i.e., ST sharing at least five out of seven allelic variants), with 188 ST accounting for 1,658 isolates (77.4%) being grouped in over 35 different CC. The remaining 119 ST, accounting for 484 isolates (22.6%), were considered singletons.

Most of the STs expressed almost exclusively one serotype. Among the most frequently isolated ST or CCs, for example, CC180 was strongly associated with serotype 3 (117 isolates; 99.2%), CC260 also with serotype 3 (70 isolates; 100%), ST989 with serotype 12F (175; 99.4%), and CC53 with serotype 8 (98; 99%). Other CCs displayed a more variable serotype expression, such as CC156 or CC63 expressing up to five different serotypes, and CC230, particularly during the early vaccine period (Table 2).

**Table 2:** Clonal complexes (CCs) and singletons with >40 isolates throughout the study period (2011–2016) and their expressed serotypes.

CC/ST	Serotype	No.EVP (%) 1064	No.EVP (%) 1078	p-value
**ST89**		**69 (6.5%)**	**107 (9.9%)**	**0.004**
	12F	68 (98.6)	107 (100)	
	8	1 (1.4%)	0	
**CC156**		**69 (6.5%)**	**106 (9.8%)**	**0.005**
	14	53 (76.8)	85 (80.2)	
	11A	7 (10.1)	14 (13.2)	
	9V	7 (10.1)	6 (5.7)	
	19A	1 (1.4)	1 (0.9)	
	24F	1 (1.4)	0	
**CC180**		**56 (5.3%)**	**62 (5.8%)**	**0.622**
	3	55 (98.2)	62 (100)	
	19F	1 (1.8)	0	
**CC53**		**39 (3.7)**	**61 (5.7)**	**0.029**
	8	37 (94.9)	61 (100)	
	13	1 (2.6)	0	
	11C	1 (2.6)	0	
**CC230**		**54 (5.1%)**	**50 (4.6%)**	**0.640**
	24B/F	47 (87.1)	49 (98.0)	
	19A	7 (13.0)	1 (2.0)	
**CC306**		**116 (10.9%)**	**47 (4.4%)**	**<0.001**
	1	115 (99.1)	44 (93.6)	
	NT	1 (0.9)	3 (6.4)	
**CC404**		**16 (1.5%)**	**43 (4.0%)**	**<0.001**
	8	16 (100)	43 (100)	
**CC433**		**46 (4.3%)**	**37 (3.4%)**	**0.288**
	22F	46 (100)	37 (100)	
**CC260**		**36 (3.4%)**	**34 (3.2%)**	**0.767**
	3	36 (100)	34 (100)	
**CC63**		**21 (2.0%)**	**27 (2.5%)**	**0.412**
	15A/B/F	16 (76.2)	23 (85.2)	
	19F	2 (9.5)	2 (7.4)	
	14	2 (9.5)	1 (3.7)	
	19A	1 (4.8)	0	
	23B	0	1 (3.7)	
**CC191**		**69 (6.5%)**	**20 (1.9%)**	**<0.001**
	7F	69 (100)	20 (100)	
**CC320**		**36 (3.4%)**	**14 (1.3%)**	**0.001**
	19A	36 (100)	14 (100)	

CC, clonal complex (eBURST); ST, Sequence type; EVP, Early vaccine period; LVP, Late vaccine period. In Italics there are depicted those serotypes included in PCV13. p-value, clone proportion compared with the total number of episodes registered in EVP (1064) and LVP (1078). %, serotype proportion compared with the number of clones in each study period.

As serotypes were frequently correlated to CCs, the evolution of CCs usually parallels serotype variation over time. We observed statistically significant decreases of the proportions of CC306, CC191 and CC320, related respectively to PCV13 serotypes 1, 7F and 19A. CC156, related to serotype 14, showed a significant increase, while CC180 and CC260, both related to serotype 3, did not show a significant change between EVP and LVP. When we looked at the CCs expressing mostly non-PCV13 serotypes, we observed significant increases in ST989, which was related mostly to serotype 12F, CC53 and CC404 which were both related in almost all cases to serotype 8 (Table 2).

Differences in clonal distribution by age-group were detected. In younger than 5 years, CC306, CC230 and CC156 were the most frequent CCs in EVP *vs* CC230, CC156 and CC97 in LVP. In children 5–17, the main clone standing out as the leader in EVP clearly was CC306 followed by ST989 and CC230 *vs* CC306, CC156 and ST989. In adults 18–64 the three major clones were CC306, ST989 and CC191 *vs* ST989, CC53 and CC56. Finally in seniors older than 65, CC180 and CC156 remained as the two main clones both in EVP and LVP apart from CC191 in EVP and ST898 in LVP.

Indication of capsular switching, i.e., a significant change of serotype expression by a CC, was observed in CC230 and CC1201, expressing both the vaccine serotype 19A, and also the non-vaccine serotypes 24F and 7C, respectively. In both cases, the expression of the vaccine serotype decreased during the study period while the proportion of non-vaccine serotypes increased. In addition, we have observed the emergence of ST6521, a double locus variant of ST156, expressing serotype 11A as a potential capsular switching involving ST156.

## Discussion

In this study we have analysed the serotype distribution, incidence and clonal composition of invasive pneumococcal strains after PCV13 introduction in 23 sites in Catalonia. Despite intermediate vaccination uptake of PCV13 in our region, a significant decrease in PCV13 IPD was observed in young children, 5–17 years group and adults aged 18–64 showing an important overall and indirect effect of this uncomplete childhood vaccination coverage. A significant decrease in high invasive disease potential serotypes, such as serotypes 1, 5, 7F or 19A [26] associated in the past with important burden of disease in our country [27], supports a beneficial effect of vaccination. A decline of these invasive serotypes owed to PCV13 vaccination has been reported worldwide by other authors [28–30].

Worryingly, we observed persistence of two PCV13 serotypes, namely serotype 14, which remains the second main serotype causing IPD in children younger than 5 years, and serotype 3, particularly frequent in older than 65. The persistence of these serotypes is related to two major sequence types: the antimicrobial multiresistant clone ST156 expressing serotype 14 and ST180 expressing serotype 3. The reasons why serotype 14 declined in PCV7 period and persisted in PCV13 period are not well known [31]. It would be important to monitor the evolution of this serotype after systematic introduction of PCV13 vaccine. Despite a decreasing trend of serotype 3 in young children, the incidence of this serotype remained unchanged overall. This serotype has been associated with PCV13 vaccine failures in Spain and Portugal [5]. Specific characteristics related to the synthesis of capsular type 3 polysaccharide and the notable thickness of the capsule produced by serotype 3 strains [32,33] could explain the lower protection of PCV13 vaccine against this serotype compared to other vaccine serotypes [33,34].

With respect to all-type IPD, a significant decline was only observed in the group of children younger than 5 year, while in elderly there was a significantly higher IPD incidence, due to more frequent non-vaccine IPD and a modest decline in PCV13 IPD. These general trends are widely observed in studies from different settings: serotypes included in the vaccine tend to decrease in all age groups, while serotypes not included in the vaccine tend to increase in all age groups, both in proportion and in incidence. The effect on all-type IPD incidence is consistently favourable in children younger than 5 years, but this positive effect decreases with age. Surveillance studies have shown positive, neutral and non-significant negative effect on all-type IPD incidence in older adults [35, 36]. The reason of these differences could be related to the valence of the vaccine included in the schedule, to the different contact patterns between young children and elderly, or the limited vaccination coverage.

Interestingly, we have observed a significant increase of non-PCV13 serotypes in adults and seniors but it was not observed in pediatric population. This situation is similar to that reported in England and Wales [37], where IPD incidence in children younger than 5 years remained stable during the first four years after PCV13 introduction. However significant serotype replacement phenomenon was reported in adults. Differences in the distribution of major serotypes by age in the pre-vaccine period could be related with this fact. In Table 1 and Fig 2 it can be observed that non-PCV13 serotypes comprise over half of the adult population whilst in the pediatric population the main leaders are the PCV13 serotypes.

Nowadays, the major serotypes causing IPD are the nonPCV13 serotypes 12F and 8. These serotypes are well recognized for their high invasive disease potential [38]. In our study, the emergence of hypervirulent serotypes 12F and 8 was correlated to the significant increase of three major clones ST989, CC404 and CC53. In LVP all ST989 strains expressed serotype 12F, while all CC404 and CC53 strains expressed serotype 8. These trends have also been described in other age-specific studies. In England and Wales, a spread of these serotypes has also been associated with a decrease in vaccination’s benefits [37]. Important outbreaks and emergence of serotype 12F expressed by different sequence types have been recently reported [39, 40]. In addition, an increased circulation of CC53 expressing serotype 8 has been reported in Canada [41], which warrants considering the inclusion these serotypes in new PCV vaccines.

In young children, the major serotype detected is the non PCV13 serotype 24F, which is also recognized for its high invasiveness potential. Unfortunately, it is not considered to be included in the new conjugate vaccine PCV15 (PCV13 plus 22F, 33F) and PCV20 (PCV15 plus 8, 10A, 11A, 12F, 15B), currently in development [42, 43]. The increase of serotype 24F in Catalonia has been associated with the multiresistant CC230 expressing this serotype [44]. Of note, CC230 was one of the major CC related with the emergence of serotype 19A after introduction of PCV7 in our country [27]. This capsular switching together with the one we observed with CC1201 highlights the highly adaptive genetics of pneumococcus, which allows it to spread highly successful clones eluding the preventing action of vaccine.

Noteworthy, the emergence of ST6521, a double locus variant of ST156, is also of concern. ST6521 has been detected expressing serotype 11A suggesting another capsular switching involving ST156. ST6521 expressing serotype 11A has been recently reported as an emerging clone causing otitis media in children of our geographical area [45]. CC156 capsular switching ability has also been reported in US, associating this clonal type with the emergence of non-vaccine serotype 35B [46]. It is important to monitor the future spread of isolates belonging to this serotype because 35B is not included in any of the current conjugate vaccines [47]. We also observed a non-significant increase in serotype 10A in children younger than 5 years and elderly. Among cases aged <5 years, serotype 10A was the third most common serotype in the LVP. Alike serotype 11A, serotype 10A is also included in PCV20.

This study has several limitations. In Catalonia, as invasive pneumococcal strains are sent on a voluntary basis to the reference laboratory, inconsistencies across years could not be ruled out resulting in over or underrepresentation of specific calendar years. To avoid this, we have only included hospitals that have systematically sent their samples. In result, we are confident of the yearly calendar representation, but on the other hand, our study population does not comprise the total Catalan population. However, referring to the observed significant drop in the overall IPD IR, our results show an overall consistency with surveillance data collected during a similar study period (2012–2016) in Catalonia [40]. Our study population includes 67% of Catalan young children and 43% of Catalan people older than 5 years. We avoided over representing data from children by presenting results stratified by age. We did not have yearly data on PCV13 coverage or on 23 valent polysaccharide vaccine (PPSV23) coverage, which may have played a role in the serotypes and clones distribution dynamics. In 1983, the 23-valent pneumococcal polysaccharide vaccine (PPSV23) was introduced in the USA. In Catalonia has been recommended for high-risk and older adults since the 2000s, reaching a coverage around 60–70% in these persons [48]. However, assuming that PPSV23 coverage was stable and that PCV13 coverage steadily increased during the study period seems reasonable. Moreover, additional data like antibiotic use, comorbidities or vaccination status for individual patients was not requested as well as possible changes in clinical and blood culture practices, which might have influenced IPD rate in our study. However, guidelines based on clinical suspicion of IPD have not substantially changed throughout the study period.

In conclusion, vaccinating children with PCV remains an effective option to prevent paediatric IPD. The persistence of PCV13 serotypes in children and modest indirect protection against vaccine serotypes causing IPD in adults indicate the importance of increasing coverage of PCV through systematic vaccination. The inclusion of more serotypes in future high-valent PCV and vaccinating old adults should be also considered.
